# Risk factors for lateral pelvic lymph node metastasis in patients with lower rectal cancer: a systematic review and meta-analysis

**DOI:** 10.3389/fonc.2023.1219608

**Published:** 2023-09-06

**Authors:** De-xing Zeng, Zhou Yang, Ling Tan, Meng-ni Ran, Zi-lin Liu, Jiang-wei Xiao

**Affiliations:** ^1^Department of Gastrointestinal Surgery, Clinical Medical College and The First Affiliated Hospital of Chengdu Medical College, Chengdu, Sichuan, China; ^2^Department of Gastrointestinal Surgery, Sichuan Provincial People’s Hospital, University of Electronic Science and Technology of China, Chengdu, China; ^3^Department of Urology, People’s Hospital Affiliated to Chongqing Three Gorges Medical College, Chongqing, China; ^4^Department of Pharmacy, Three Gorges Hospital Affiliated to Chongqing University, Chongqing, China

**Keywords:** rectal cancer, lateral pelvic lymph node metastasis, risk factors, meta-analysis, LPLN

## Abstract

**Background and objective:**

Lateral pelvic lymph node (LPLN) metastasis is one of the prominent reasons for local recurrence (LR) in patients with rectal cancer (RC). The evaluation criteria of lateral lymph node dissection (LLND) for patients in eastern (mainly in Japan) and western countries have been controversial. The aim of this study was to analyse the risk factors for LPLN metastasis in order to guide surgical methods.

**Methods:**

We searched relevant databases (Embase (Ovid), Medline (Ovid), PubMed, Cochrane Library, and Web of Science) for articles published between 1 January 2000 and 05 October 2022 to evaluate the risk factors for LPLN metastasis in patients with RC in this meta-analysis.

**Results:**

A total of 24 articles with 5843 patients were included in this study. The overall results showed that female sex, age <60 years, pretherapeutic CEA level >5 ng/ml, clinical T4 stage (cT4), clinical M1 stage (cM1), distance of the tumour from the anal verge (AV) <50 mm, tumour centre located below the peritoneal reflection (Rb), short axis (SA) of LPLN ≥8 mm before nCRT, short axis (SA) of LPLN ≥5 mm after nCRT, border irregularity of LPLN, tumour size ≥50 mm, pathological T3-4 stage (pT3-4), pathological N2 stage (pN2), mesorectal lymph node metastasis (MLNM), lymphatic invasion (LI), venous invasion (VI), CRM (+) and poor differentiation were significant risk factors for LPLN metastasis (P <0.05).

**Conclusion:**

This study summarized almost all potential risk factors of LPLN metastasis and expected to provide effective treatment strategies for patients with LRC. According to the risk factors of lateral lymph node metastasis, we can adopt different comprehensive treatment strategies. High-risk patients can perform lateral lymph node dissection to effectively reduce local recurrence; In low-risk patients, we can avoid overtreatment, reduce complications and trauma caused by lateral lymph node dissection, and maximize patient survival and quality of life.

## 1 Introduction

Colorectal cancer (CRC) is the third most common malignant tumour in the world. In 2020, it was ranked as the fourth leading cause of cancer death, second to lung cancer (1), and its burden is estimated to increase by 60% to more than 2.2 million new cases and 1.1 million cancer deaths by 2030 (2).

Local recurrence (LR) of RC is still a serious clinical problem that is related to low survival and high incidence rates. It diffuses through the superior lymphatic drainage of the inferior mesenteric artery as well as the lateral lymphatic drainage of the internal iliac artery outside the rectum (3, 4). Lateral pelvic lymph node (LPLN) metastasis is considered the main cause of LR in patients with low rectal cancer (LRC) (5–7). Several studies verified that the incidence of LPLN metastasis in patients with LRC was approximately 15% (8), while the incidence of stages T3 and T4 exceeded 20% (9, 10). Klusters M et al. assumed that lymph and tumour cells wound flow into the LPLN system when the tumour is crushed during surgical resection. In addition, the LPLN system was left untouched during standard TME, and partial damage during rapid dissection of the lateral ligament led to lateral positive lymphatic residue. Finally, lymph converged in the presacral area and flowed into serum, which might have led to local tumour recurrence (11). It is urgent to find the relevant risk factors for LPLN metastasis. However, recent studies have shown that lateral recurrence has become the most common recurrence mode, accounting for up to 50%~82.7%. Lateral recurrence after rectal cancer surgery has been heavily discussed and is a barrier to prevention and treatment of colorectal surgery (6).

However, there has been no meta-analysis to clarify the risk factors for LPLN metastasis in patients with LRC to date. We included all significantly relevant articles to compile this meta-analysis to further guide the treatment of rectal cancer patients with suspected LPLN metastasis. It can guide us to identify which patients with rectal cancer need lateral lymph node dissection to reduce the risk of local recurrence.

## 2 Materials and methods

### 2.1 Literature search

Studies published up to 05 October 2022 were identified by searching Embase (Ovid), Medline (Ovid), PubMed, Cochrane Library, and Web of Science. No regional restriction was imposed. Articles were confined to human studies published in English. The search algorithms consisted of Medical Subject Headings (MeSH) and free text terms, including the following: “Rectal cancer”, “Lateral pelvic lymph node metastasis”, and “risk factor”. Eligible literature was identified by reading the included relevant articles.

### 2.2 Article selection

Inclusion criteria: (1) participants: rectal cancer patients with clinically suspected LPLN metastasis; (2) intervention: pathological examination confirmed positive metastasis of LPLN; (3) comparison: pathological examination confirmed negative metastasis of LPLN; (4) outcome measures: report at least one of the endpoints listed in **Table 1**; (5) study design: randomized controlled trials, prospective or retrospective cohort and case-control studies. Studies were excluded if: (1) they were reviews, case reports, conference articles or unrelated studies (the article did not contain rectal cancer, lymphatic metastasis, or risk factor analysis); (2) the metastatic lymph node was not LPLN; and (3) no outcome measures of interest were reported.

**Table 1:** Main characteristics of the selected studies.

Reference	Journal	Country	N	LPLN (+) rate	Age	Operation method	Outcome
Abe 2022 (12)	World Journal of Surgical Oncology	Japan	67	26.9%	LPLN(+): 66.5 (47-83) LPLN(-): 65 (33-78)	laparoscopy/open	1, 3, 10a, 12, 13, 14, 15, 17
Dev 2018 (13)	Indian Journal of Surgical Oncology	India	43	20.9%	/	/	1, 4, 7a, 9, 10a, 10b, 11a, 13, 17
E. Agger 2021 (14)	International Journal of Colorectal Disease	Sweden	344	8.7%	/	/	1, 3, 10a, 10c, 14
Fujita 2009 (15)	International Journal of Colorectal Disease	Japan	210	22.4%	/	/	1, 2, 4, 8, 9, 10a, 11a, 12, 13, 14, 15, 17
Hiyoshi 2019 (16)	International Journal of Clinical Oncology	Japan	78	11.5%	62.8 (19–80)	laparoscopy/open	1, 3, 4, 8, 10c, 13, 17
Ishibe 2020 (17)	International Journal of Colorectal Disease	Japan	458	15.5%	63 (28–86)	open	1, 4, 7b, 9, 13, 17
Iwasa 2021 (18)	International Journal of Colorectal Disease	Japan	102	19.6%	64 (30–82)	/	3, 4, 7a, 8, 10c, 16
Kawai 2021 (19)	Disease Of The Colon & Rectum	Japan	279	9.3%	64 (32–86)	/	1, 2, 6a, 7b, 10a, 10c
Kim 2007 (6)	Annals of Surgical Oncology	Korea	366	6.6%	57 (27–83)	/	1, 4, 6b, 7a, 9, 10a, 16, 17
Kim 2018 (20)	PLOS ONE	Korea	57	40.4%	57 (50–67)	/	1, 4, 5a, 5b, 6a, 6b, 7a, 9, 10a, 11a, 11b, 12, 14, 15, 17
Komori 2018 (21)	European Journal of Surgical Oncology	Japan	328	7.3%	/	/	1, 2, 6a, 7a, 8, 9, 11b, 17
Lim 2013 (22)	International Journal of Colorectal Disease	Korea	67	40.0%	/	/	1, 8, 10a, 10b, 11a, 11b, 12, 15, 16, 17
Malakorn 2019 (23)	Disease Of The Colon & Rectum	America	64	51.6%	/	/	1, 6b, 11a, 11b, 12, 15
Nakanish 2020 (24)	Annls Surg Oncology	Japan	247	28.7%	60 (49–67)	/	1, 4, 10a, 11a, 17
Ogawa 2016 (25)	International Journal of Colorectal Disease	Japan	394	21.3%	64 (16–87)	/	1, 10c, 11a, 13, 17
Oh 2014 (26)	Annls Surg Oncology	Korea	66	33.3%	58.5 (31-82)	laparoscopy/open	1, 9, 10a, 10b
Park 2018 (27)	journal of surgical research	Korea	99	32.3%	/	/	1, 4, 6b, 7a, 10a, 17
Sekido 2019 (28)	Surgery Today	Japan	60	20.0%	60 (19–77)	/	1, 2, 6b, 7b, 10a, 17
Sugihara 2006 (7)	Dis Colon Rectum	Japan	1977	6.5%	/	/	1, 8, 11a, 12, 13, 14, 17
Wang 2019 (29)	Colorectal Disease	China	76	17.1%	54.33 ± 10.03	laparoscopy/open	1, 2, 4, 6b, 7a, 17
Wang 2020 (30)	Journal of Gastrointestinal Surgery	Japan	215	18.6%	/	laparoscopy/open	1, 3, 4, 11a, 11b, 14, 17
Wu 2007 (31)	World Journal of Gastroenterology	China	96	14.6%	65 (25-86)	/	1, 2, 4, 9, 12, 14, 17
Yang 2021 (32)	Techniques in Coloproctology	China	77	28.6%	54 (25–89)	laparoscopy/open	1, 2, 3, 4, 6a, 7a, 10a, 10b, 11a, 11b, 17
Zhou 2021 (33)	BMC Surgery	China	73	20.5%	55.8 ± 10.4	laparoscopy/open	1, 2, 4, 5a, 7a, 11a, 12, 14, 15, 17

LPLN, lateral pelvic lymph node; Outcome: 1 gender, 2 age, 3 preoperative therapy, 4 pre-therapy CEA (ng/ml), 5a border irregularity of LPLN, 5b mixed signal intensity of LPLN, 6a Short axis before CRT (mm), 6b Short diameter after CRT (mm), 7a distance of the tumor from the anal verge (50mm), 7b distance of the tumor from the anal verge (40mm), 8 tumor location, 9 tumor size (mm), 10a cT, 10b cN, 10c cM, 11a pT, 11b pN, 12 lymphatic invasion, 13 MLNM: mesorectal lymph node metastasis, 14 venous invasion, 15 perineural invasion, 16 CRM, 17 differentiation.

### 2.3 Outcomes of interest

We tried to screen all comparable data of the included articles as fully as possible. When a certain indicator contains data with more than 2 articles, it is considered as “Outcomes of Interest”. The indicators were as follows: Sex, age, pretherapeutic CEA level (ng/ml), border irregularity of LPLN, mixed signal intensity of LPLN, short axis of LPLN before CRT (mm), short axis of LPLN after CRT (mm), distance of the tumour from the AV (50 mm or 40 mm), tumour location, tumour size (mm), cT, cN, cM, pT, pN, LI, MLNM, VI, PI, CRM and differentiation.

### 2.4 Data extraction and outcome measures

Two authors (ZDX and TL) independently screened all the included studies and extracted the relevant data. Divergence of views was resolved through discussion between the authors. When consensus could not be reached, the third author (RMN) was consulted, and a discussion ensued until a consensus was reached. The following relevant information was extracted from all the included studies: reference, journal, country, number of patients, LPLN (+) rate, age, operation method and endpoints.

### 2.5 Study quality assessment

The quality of the enrolled studies was evaluated by two authors independently using the Newcastle Ottawa Scale (NOS), with a maximum of nine points per study (34). Studies with a score <6 were considered low-quality studies and excluded. For this systematic review, we adhered to the Meta-analysis of Observational Studies guidelines and the Reporting Items for Systematic reviews and Meta-Analysis (PRISMA) statement (35).

### 2.6 Statistical analysis

We used RevMan 5.4 software from the Cochrane Collaboration for all statistical analyses. Odds ratios (ORs) with 95% confidence intervals (CIs) were assessed to analyse dichotomous variables. p of Q test >0.1 and I^2^ < 50% illustrated a lack of heterogeneity, and in this case, the pooled estimate was calculated by a fixed effects model. Otherwise, when p of Q test <0.1 or I^2^ >50%, a random effects model was adopted. A leave-one-out sensitivity analysis was performed by excluding one study back and forth to confirm that our results were not driven by any single trial. Publication bias was assessed by visual inspection of the symmetry of a funnel plot. The level of significance was defined as p <0.05 (test for heterogeneity was set at p <0.1).

## 3 Results

### 3.1 Study selection and characteristics

**Figure 1:**
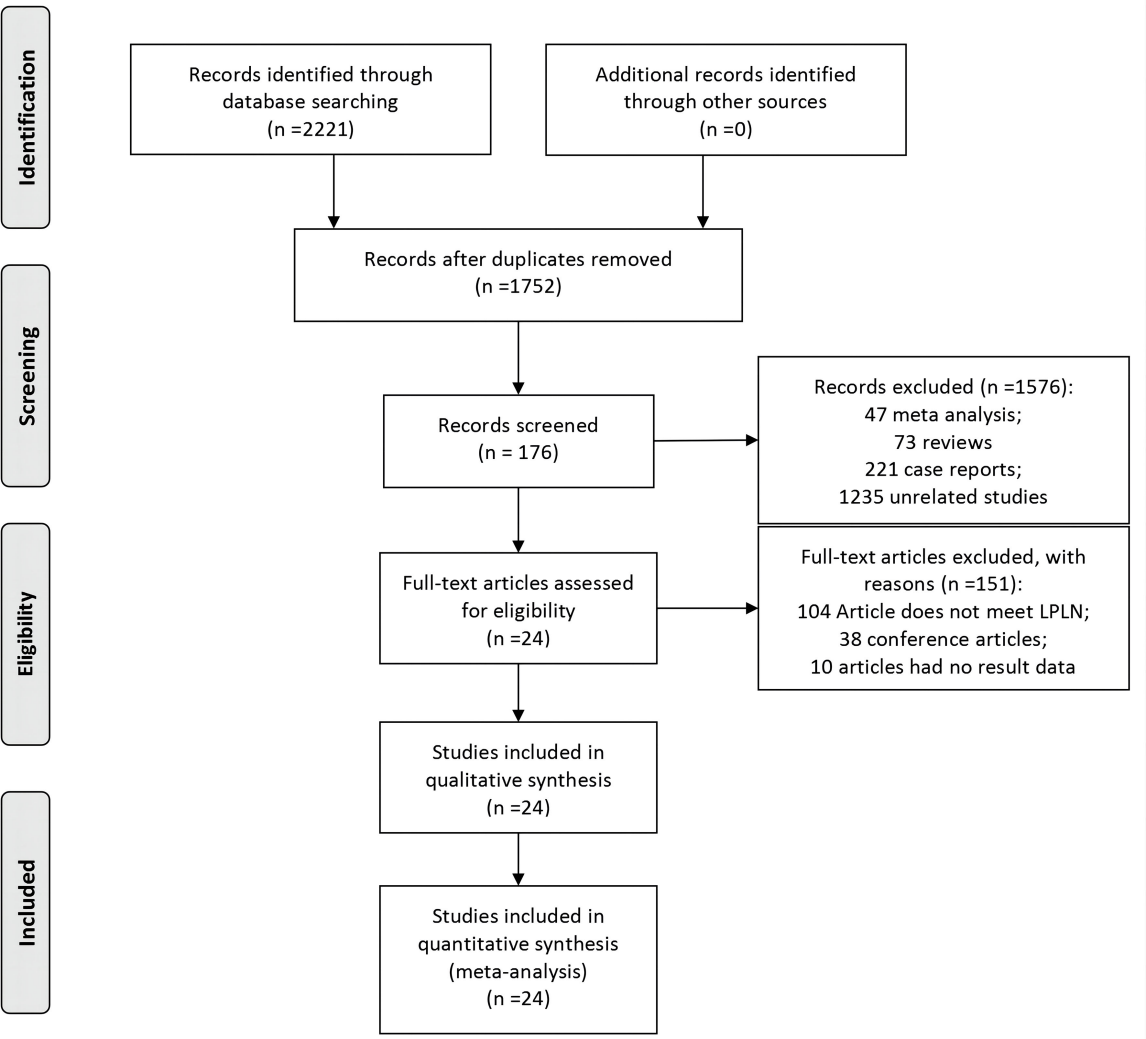
Inclusion and exclusion criteria chart.

**Figure 2:**
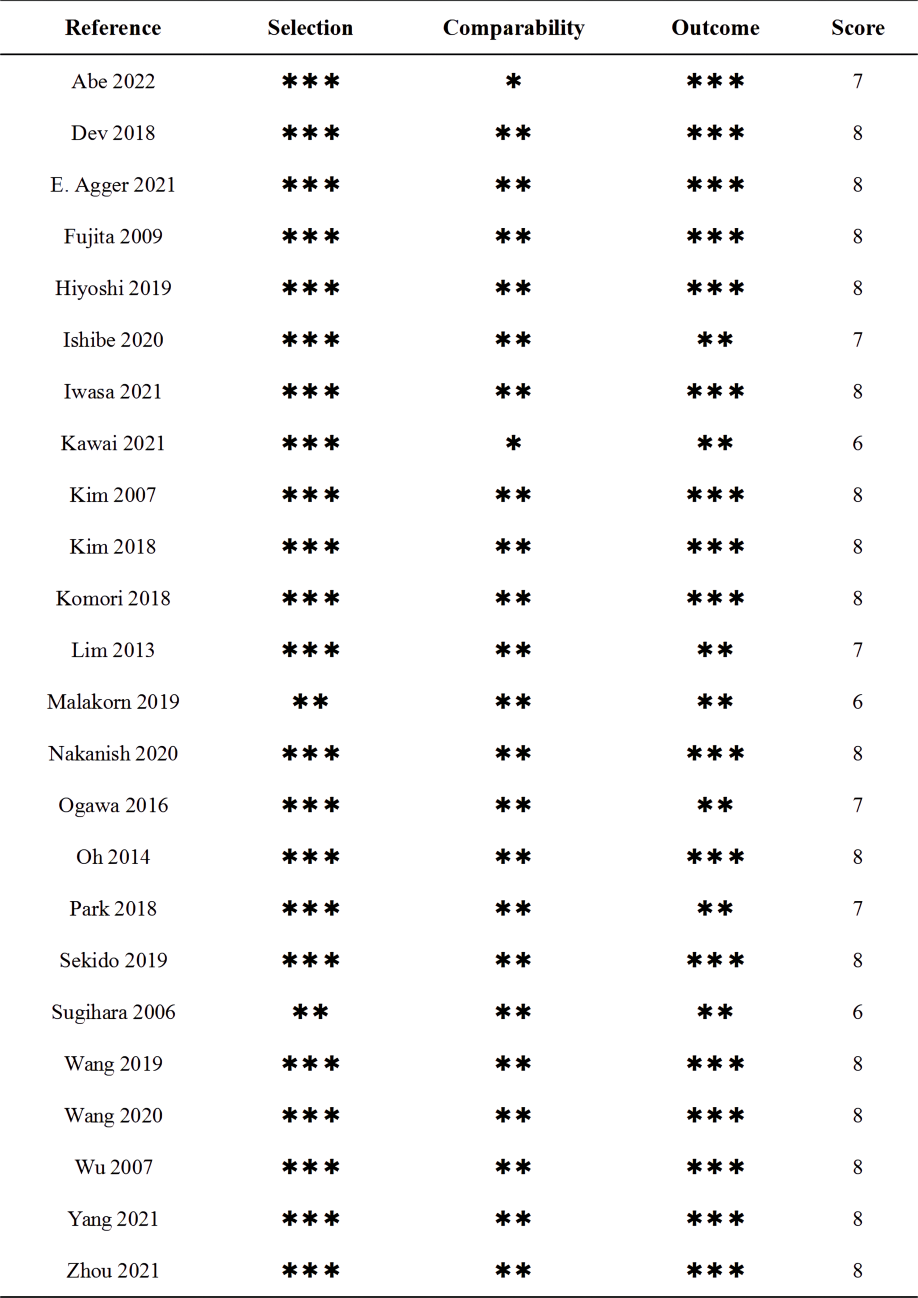
The NOS scores of studies. The * represent the various scores of the NOS scale.

The flow chart for the inclusion of articles is shown in **Figure 1**. A total of 24 studies were eventually included in the quantitative synthesis by screening databases through search strategies in advance (6, 7, 12–33). The baseline characteristics and lymph details of the studies are displayed in **Table 1**. A total of 24 retrospective articles with 5843 patients were included in this study, of which the LPLN-positive rate was between 6.5% and 51.6%. Most articles were reported in East Asia (12 in Japan, 5 in Korea, 4 in China and 1 in India), but 2 were reported in Western countries (1 in Sweden and the other in America). The NOS scores of the studies are displayed in **Figure 2**, and all studies scored 6 points or higher.

### 3.2 Outcomes of baseline characteristics

**Figure 3:**
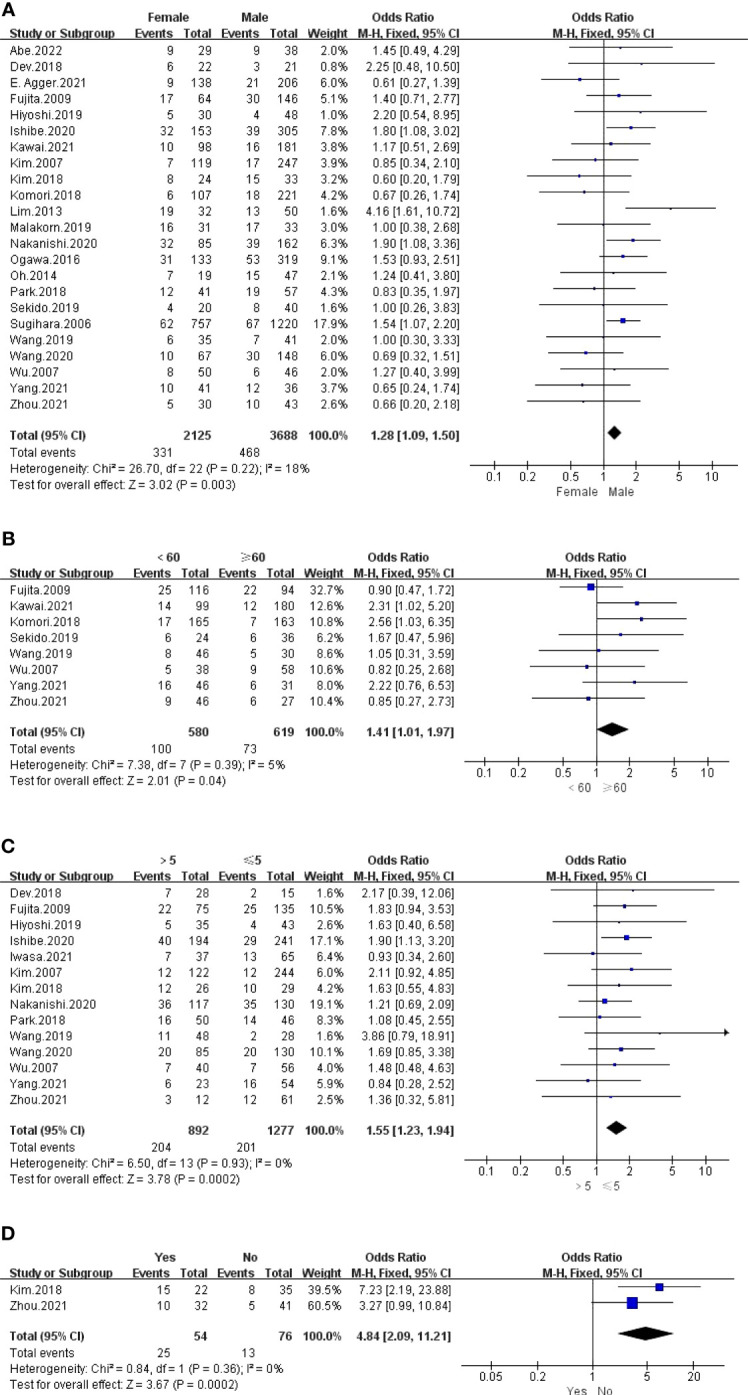
**(A)**. Gender; **(B)** Age; **(C)** Pre-therapy CEA level; **(D)** Border irregularity of LPLN.

The outcomes are summarized in **Figures 3A, B**. For all outcomes, low statistical heterogeneity existed between the studies, and the fixed effects model was used. The pooled results showed a significantly higher risk of LPLN metastasis in females (OR: 1.28, 95% CI: 1.09-1.50, I^2 ^= 18%, P =0.003) and age <60 years (OR: 1.41, 95% CI: 1.01-1.97, I^2 ^= 5%, P =0.04).

### 3.3 Preoperative examination results

#### 3.3.1 Pretherapy CEA level (ng/ml)

The outcome is listed in **Figure 3C**. No statistical heterogeneity existed between the studies; thus, the fixed effects model was used. Graphics demonstrated that a pretherapeutic CEA level >5 ng/ml was strongly associated with LPLN metastasis (OR: 1.55, 95% CI: 1.23-1.94, I^2 ^= 0%, P =0.0002).

#### 3.3.2 Tumour border and signal characteristics on MRI

The outcomes are listed in **Figures 3D** and **S1**. Pooled results revealed a significantly higher risk of LPLN metastasis with border irregularity on MRI (OR: 4.84, 95% CI: 2.09-11.21, I^2 ^= 0%, P =0.0002). Regarding tumour signal characteristics, the random effects model was used due to obvious statistical heterogeneity, photographs seemed to not affect LPLN metastasis (OR: 3.98, 95% CI: 0.77-20.56, I^2 ^= 76%, P =0.10).

#### 3.3.3 SA of LPLN on MRI/CT (mm)

**Figure 4:**
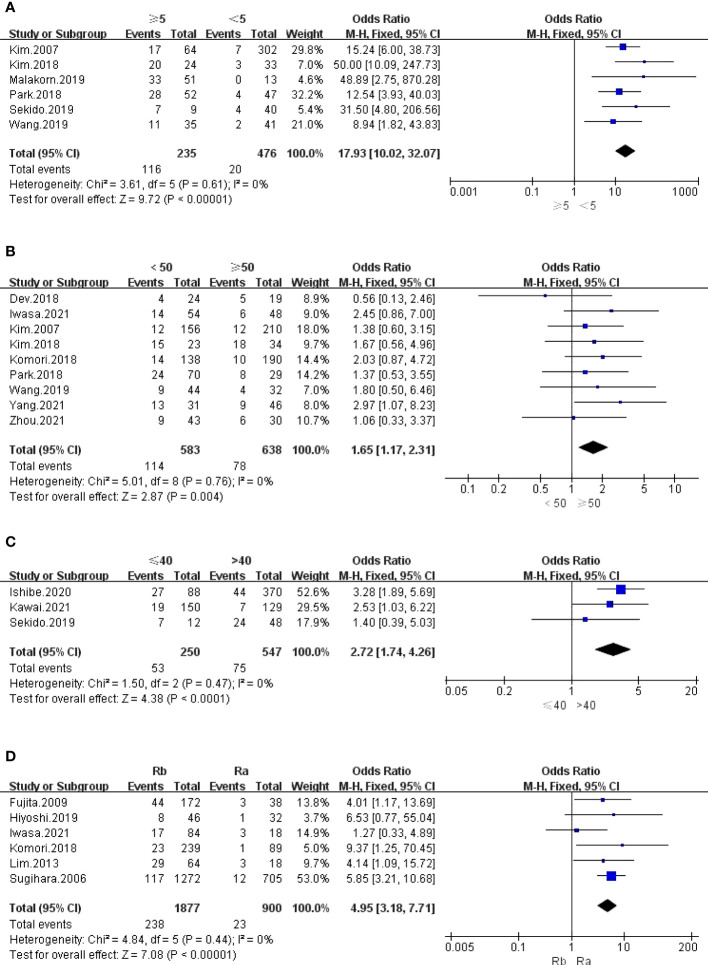
**(A)** LPLN ≥5mm after nCRT; **(B)** Distance of the tumor from the AV <50mm; **(C)** Distance of the tumor from the AV ≤40mm; **(D)** Tumor center located Rb.

The outcomes are summarized in **Figures 4A** and **S2**. The fixed effects model was used because no statistical heterogeneity existed in the SA of LPLN ≥5 mm after nCRT, while the random effects model was used because obvious statistical heterogeneity existed in the SA of LPLN ≥8 mm before nCRT. Overall, the results showed that both SA of LPLN ≥5 mm after nCRT (OR: 17.93, 95% CI: 10.02-32.07, I^2 ^= 0%, P <0.00001) and SA of LPLN ≥8 mm before nCRT (OR: 9.33, 95% CI: 3.51-24.83, I^2 ^= 68%, P <0.00001) proved to be hazard factors for LPLN metastasis.

#### 3.3.4 Tumour location and size

**Figure 5:**
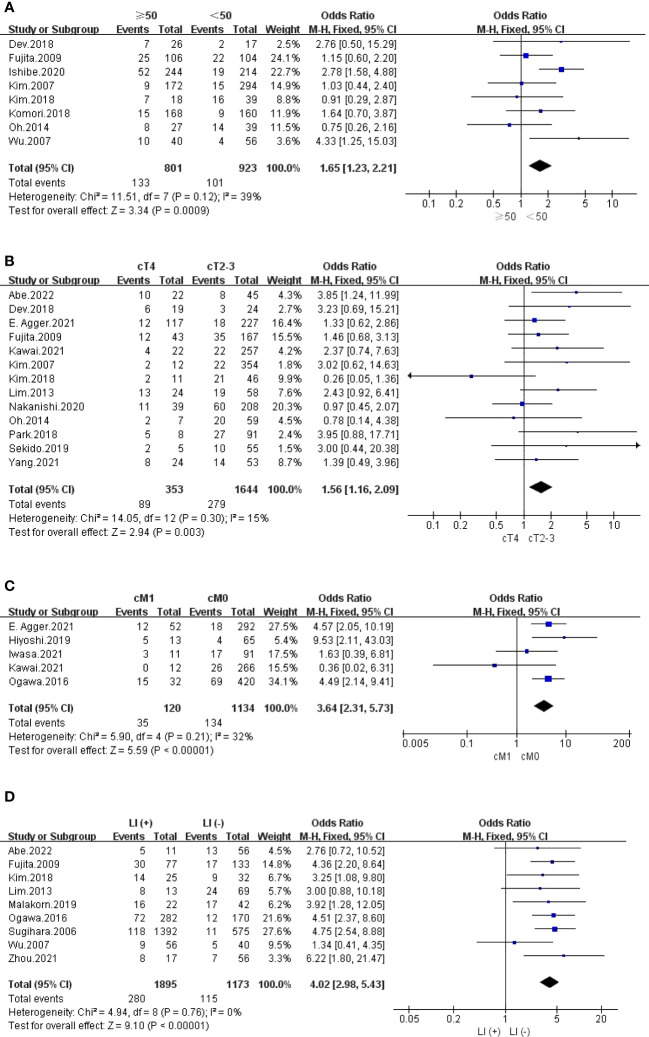
**(A)** Tumor size ≥50 mm; **(B)** cT4; **(C)** cM1; **(D)** LI.

The outcomes are summarized in **Figures 4B–D** and **5A**. The fixed effects model was used owing to low statistical heterogeneity. Overall, the results showed a significantly higher risk of LPLN metastasis in the distance of the tumour from the AV <50 mm (OR: 1.65, 95% CI: 1.17-2.31, I^2 ^= 0%, P =0.004) or ≤40 mm (OR: 2.72, 95% CI: 1.74-4.26, I^2 ^= 0%, P <0.0001), tumour centre located Rb (OR: 4.95, 95% CI: 3.18-7.71, I^2 ^= 0%, P <0.00001), and tumour size ≥50 mm (OR: 1.65, 95% CI: 1.23-2.21, I^2 ^= 39%, P =0.0009).

#### 3.3.5 cTNM stage

The outcomes are summarized in **Figures 5B, C** and **S3**. The fixed effects model was used owing to low statistical heterogeneity. Pooled results revealed that both cT4 (OR: 1.56, 95% CI: 1.16-2.09, I^2 ^= 15%, P =0.003) and cM1 (OR: 3.64, 95% CI: 2.31-5.73, I^2 ^= 32%, P <0.00001) were hazard factors for LPLN metastasis. However, cN2-3 did not seem to affect LPLN metastasis (OR: 1.09, 95% CI: 0.61-1.93, I^2 ^= 0%, P =0.77).

### 3.4 Postoperative examination results

#### 3.4.1 pTN stage

The outcomes are summarized in **Figures S4, 5**. The random effects model was used because obvious statistical heterogeneity existed in pT stage, while the fixed effects model was used because no statistical heterogeneity existed in pN stage. Overall, the results showed that both pT3-4 (OR: 2.81, 95% CI: 1.83-4.30, I^2 ^= 59%, P <0.00001) and pN2 (OR: 7.61, 95% CI: 4.88-11.85, I^2 ^= 0%, P <0.00001) were conspicuous hazard factors for LPLN metastasis.

#### 3.4.2 Invasion

**Figure 6:**
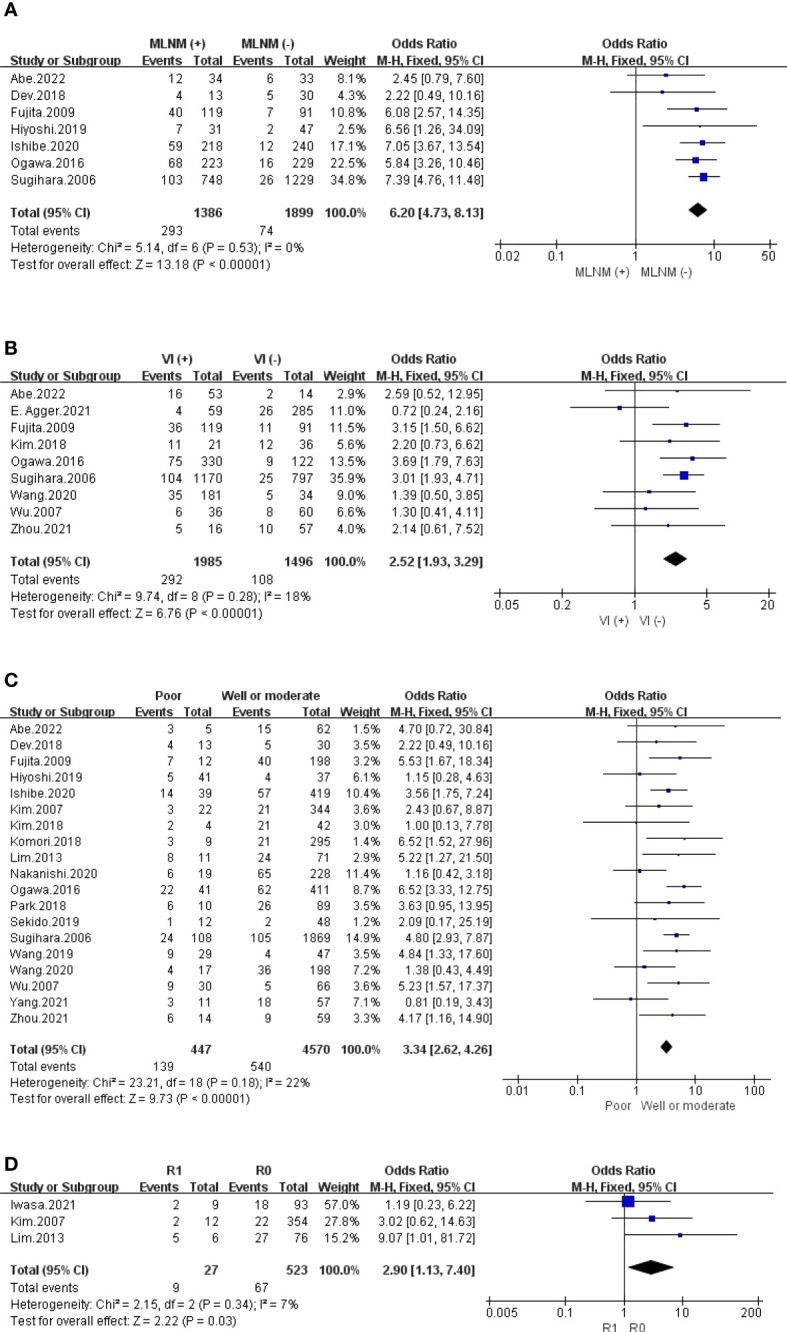
**(A)** MINM; **(B)** VI; **(C)** Differentiation; **(D)** CRM.

The outcomes are summarized in **Figures 5D**, **6A, B** and **S6**. Regarding LI, MLNM and VI, the fixed effects model was used owing to low statistical heterogeneity. Overall, the results showed a significantly higher risk of LPLN metastasis in LI (OR: 4.02, 95% CI: 2.98-5.43, I^2 ^= 0%, P <0.00001), MLNM (OR: 6.20, 95% CI: 4.73-8.13, I^2 ^= 0%, P <0.00001) and VI (OR: 2.52, 95% CI: 1.93-3.29, I^2 ^= 18%, P <0.00001). While the random effects model was used because obvious statistical heterogeneity existed in the PI, photographs seemed to not affect LPLN metastasis (OR: 1.45, 95% CI: 0.86-2.45, I^2 ^= 56%, P =0.17).

#### 3.4.3 Differentiation and CRM

**Figure 7:**
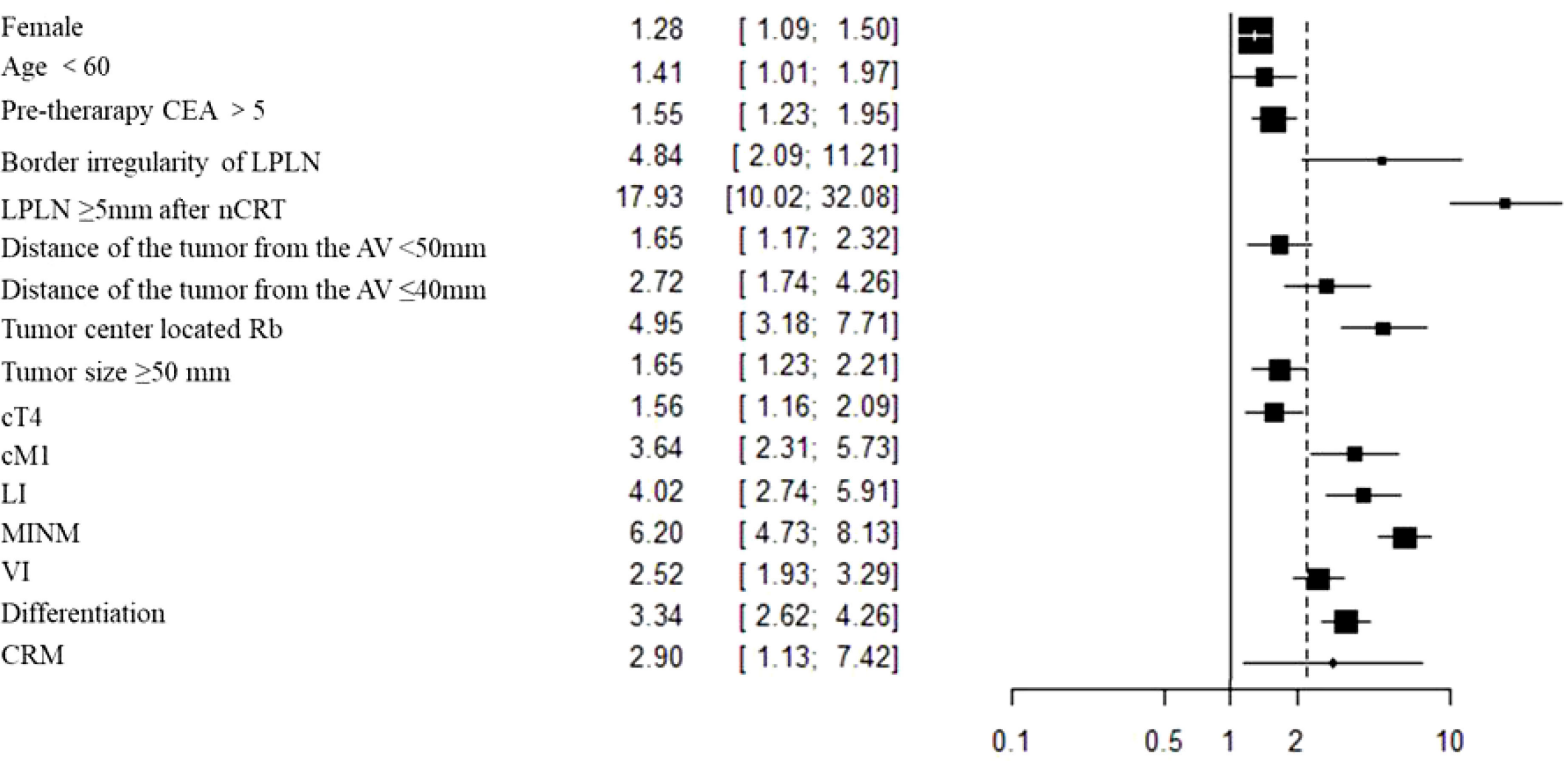
The total valuable variables as possible risk factors for LPLN metastasis.

**Figure 8:**
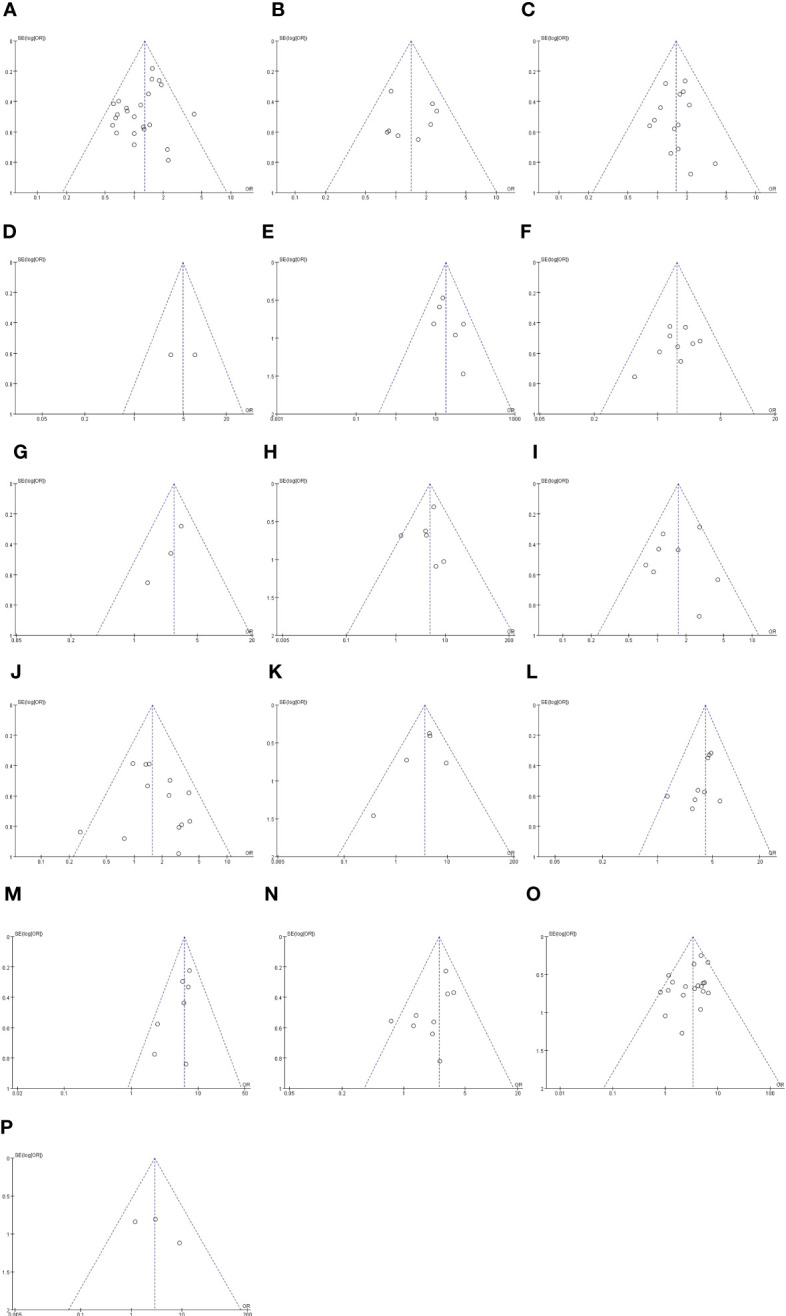
Funnel plot of publication bias in the meta-analysis. **(A)** Gender. **(B)** Age **(C)** Pre-therapy CEA level. **(D)** Border irregularity of LPLN. **(E)** SA of LPLN ≥5mm after nCRT. **(F)** The distance of the tumor from the AV <50mm. **(G)** The distance of the tumor from the AV ≤40mm. **(H)** Tumor center located Rb. **(I)** Tumor size ≥50 mm. **(J)** cT. **(K)** cM. **(L)** LI. **(M)** MLNM. **(N)** VI. **(O)** Differentiation. **(P)** CRM.

The outcomes are summarized in **Figures 6C, D**. The fixed effects model was used owing to low statistical heterogeneity. Overall, the results showed a significantly higher risk of LPLN metastasis in poor differentiation (OR: 3.34, 95% CI: 2.62-4.26, I^2 ^= 22%, P <0.00001) and R1 (OR: 2.90, 95% CI: 1.13-7.40, I^2 ^= 7%, P =0.03). The total valuable variables as possible risk factors for LPLN metastasis were summarized in **Figure 7**. The funnel plot of publication bias which included various indicators were listed in **Figure 8**.

## 4 Discussion

Surgical treatment is the main treatment for rectal cancer, in which radical resection and regional lymph node dissection are the key to success. The special drainage characteristics of rectal cancer lymph nodes determine the extent of lymph node dissection. The rectal lymphatic drainage area is distributed along the medial space of the obturator foramen of the internal iliac artery, and once metastasis occurs, it will spread upwards, laterally and downwards. It is worth noting that lateral lymph node metastasis is a metastatic pathway of low rectal cancer. Chemoradiotherapy has a poor effect and affects the prognosis of patients with rectal cancer. Previous literature has reported that LPLN metastasis is the main cause of LR in patients with LRC. Postoperative LR is a serious complication in patients with LRC that leads to pain, ureteral and intestinal obstruction, fistula and inflammation and significantly reduces the quality of life of patients. The prevention of LR is crucial because of the poor treatment effect when LR develops (36). Lateral lymph node metastasis is a common problem in the diagnosis and treatment of low rectal cancer, but there is still controversy between Eastern and Western scholars on whether TME should be combined with lateral lymph node dissection for middle and low rectal cancer (37). The studies and literature of Japanese scholars have confirmed that the effect of lateral lymph node dissection is affirmative, which can significantly reduce the local recurrence rate and significantly improve the 5-year survival rate. At present, lateral lymph node dissection has become a standard procedure in Japan. However, this procedure is not widely accepted in Western countries. The treatment for advanced rectal cancer in Europe and America is preoperative neoadjuvant chemoradiotherapy (nCRT)+TME as a standard treatment strategy based on the explanation that LPLN is considered to be a systemic disease as well as unresectable by the TME procedure alone (38). In addition, several studies have authenticated that nCRT can reduce the rate of local recurrence (39, 40). However, Ogura et al. refuted that it is still a problem with the treatment of nCRT before TME and cleared the lower proportion of local recurrence when TME was combined with LLND (41).

In recent years, on account of recognition of local disease rather than a systemic disease about LPLN (8, 42, 43), the Japanese Society for Cancer of the Colon and Rectum recommends the LLND procedure for advanced LRC, especially located below the peritoneal reflection (Rb), which is able to reduce the rate of LPLN metastasis (44), but complications such as longer operation time, higher blood loss and sexual dysfunction occur sequentially (8, 45–47). Consequently, the TME+LLND group was compared with the TME+nCRT group, and Kusters et al. found that the local recurrence rate in both groups was lower than that of the TME alone group, although there was no significant difference (48). In contrast, J.S. Williamson et al. supported the point of views that LLND, especially internal iliac lymph node metastasis, should be considered a resectable local disease and that enlarged lymph nodes that do not respond to nCRT should be surgically dissected (49).

We performed a meta-analysis to identify the risk factors for LPLN metastasis and to provide a more scientific and accurate evaluation index for lateral lymph node dissection. Our results showed that female sex, age <60 years, pretherapeutic CEA level >5 ng/ml, cT4, cM1, distance of the tumour from the AV <50 mm, tumour centre located Rb, SA of LPLN ≥8 mm before nCRT, SA of LPLN ≥5 mm after nCRT, border irregularity of LPLN, tumour size ≥50 mm, pT3-4, pN2, MLNM, LI, VI, CRM (+) and poor differentiation were risk factors for LPLN metastasis.

Similar to previous studies (42, 50, 51), our results showed that female sex was independently associated with LPLN metastasis, potentially owing to the anatomical difference between the male and female pelvis (52). Age <60 years was risk factor as well, because younger patients had the higher basal metabolic rate, and the faster the tumor progression, the higher rate of LPLN metastasis. In addition, a pretherapeutic CEA level >5 ng/ml was associated with LPLN metastasis as well.Elevated CEA levels often indicate a later tumour stage and a greater risk of lateral lymph node metastasis. Similarly, tumour size ≥50 mm was related to LPLN metastasis because the larger the tumour diameter, the greater the depth of invasion, and the higher the probability of LPLN metastasis. Additionally, lower tumour location was also related to LPLN metastasis; however, no accurate standard was determined. Nine studies used AV =50 mm, while three studies used 40 mm as the critical level. The other 6 studies used peritoneal reentry as the cut-off. Anatomically, compared with the tumour centre located at the Ra, the lymphatic drainage of the tumour centre located at the Rb was more complex (53), In addition, the internal iliac and obturator lymph nodes were the most common LPLN metastasis pathways, which were located at the Rb (54, 55). The lower the tumour location, the more drainage to the lateral lymph node region, and thus the higher the probability of LPLN metastasis.

Several studies showed that there was no significant difference between the sensitivity and specificity of CT and MRI in the diagnosis of LPLN metastasis (21, 23). A SA of the LPLN ≥8 mm before CRT was a significant risk factor for LPLN metastasis; however, there was no standard for the selection of cut-off for lymph node diameter. Some studies increased the cut-off to 7 mm (25, 31, 41), which could fully delaminate transverse local recurrence (41). Fujita et al. even advanced the cut-off to 5 mm (23, 29). More researchers predicted the risk of LPLN metastasis through the ROC curve area, and the size of the lymph node corresponding to the largest area was selected as the cut-off (26, 27, 32, 52). Although the standard is not unified, most studies set the critical value of lymph node size before nCRT as 8 mm. The results of our data analysis support that a pre-CRT SA of LPLN ≥8 mm is a significant risk factor for LPLN metastasis. Similarly, our results also showed that the SA of LPLN ≥5 mm after nCRT was significantly related to LPLN metastasis. Most of the included articles suggested 5 mm as the cut-off, except that Kawai et al. suggested 8 mm (17) and Zhou et al. suggested 7 mm (15), because 100% sensitivity was observed for a size ≥ 5 mm after nCRT to predict LPLN metastasis (18). Therefore, a SA ≥5 mm in the remaining LPLN after nCRT should be one of the clear signals of LPLN metastasis. In general, both before and after nCRT, our results showed that LPLN enlargement was significantly related to LPLN metastasis. In addition to lymph node enlargement, the specific imaging features of lymph node metastasis are also very important. Notably, the morphology of lymph nodes on MRI showing irregular boundaries and mixed signal intensity is often suggestive of LPLN metastasis, and our analysis of results also demonstrates that the irregular boundaries of LPLN is risk factor for lateral lymph node metastasis, which is consistent with previous research results (56). Some studies even found that it could improve the prediction ability of MRI for LPLN metastasis in place of lymph node size (57). Regarding the depth of cancer invasion, Wang et al. considered it an important indicator for LPLN metastasis assessed by preoperative diagnostic imaging (28). Our results showed that patients with cT4 stage were more likely to have LPLN metastasis than those with cT2-3 stage; equally, patients with cM1 stage were more prone to have LPLN metastasis than patients with cM0. Rectal lymphatic vessels arise from the lamina propria of the rectal mucosa in anatomy; thus, the percentage of lymph node metastasis is approximately 8-15% in patients with early RC (58, 59). When the tumour invades the submucosa, cancer cells have more opportunity to spread through the lymphatic vessels, leading to LPLN metastasis. In addition, the deeper the infiltration, the higher the probability of LPLN metastasis. The overall study shows that the risk of LPLN metastasis is closely related to the clinical stage of the tumour, and the later the clinical stage of the tumour, the higher the risk of LPLN metastasis.

Current studies have shown that lymphatic, venous and perineural invasions are risk factors for LR of RC, with 1 recurrence confirmed by histopathological examination in every 4 to 5 patients (60, 61). Our results showed that LI (including MLNM) and VI were strong predictors of LPLN metastasis. It is clear that LI (including MLNM) and VI indicate a later stage of the tumor and an increased risk of lateral lymph node metastasis. In addition, compared with well or moderate differentiation, our results showed that poor differentiation was a risk factor for LPLN metastasis. It is obvious that poorly differentiated carcinoma has stronger invasive and metastatic abilities and is more likely to have distant metastasis, so poorly differentiated RC is more likely to have lateral lymph node metastasis. Echoing previous reports, compared with tubular and papillary adenocarcinoma, mucinous and signet ring adenocarcinoma that did not respond to radiotherapy had a higher probability of LPLN metastasis (3, 7, 62). In addition, we also analysed whether a positive circumferential margin was a risk factor for LPLN metastasis. The results showed that a positive circumferential margin (R1) was also a risk factor for LPLN metastasis. It was obvious that RC patients with R1 were often in a later clinical stage and more likely to develop lateral lymph node metastasis.

Currently, more methods of predicting LPLN metastasis are being developed. However, it is necessary to explore better techniques to help surgeons make more accurate judgements. Dev et al. proposed a risk stratification nomogram based on important predictors of LPLN metastasis to comprehensively evaluate and guide treatment (20). Miyake et al. used a novel one-step nucleic acid amplification (OSNA) assay to calculate LPLN metastasis targeting lymph node micrometastasis with 100% sensitivity and 86% specificity, which was significantly higher than that of CT and MRI (63). Iwasa et al. proved that the presence of the middle rectal artery (MRA) assessed by ceMRI could accurately predict bilateral LPLN metastasis (including micrometastasis) (25). Abe et al. proved that extramural venous invasion on MRI (MRI-EMVI) was independently related to LPLN metastasis and proposed that it could more accurately predict LPLN metastasis combined with lymph node size (12). As we mentioned above, treatments for LPLN metastasis of LRC differ between eastern and western countries. We supported that combining the advantages of both treatments, developing strengths and avoiding weaknesses, may achieve an unprecedented effect. We recommend patients with the following risk factors: Age <60 years, female, elevated CEA level, large tumor volume, low distance from anal margin, enlarged lymph nodes with irregular enhancement (especially SA of LPLN ≥8 mm before nCRT, SA of LPLN ≥5 mm after nCRT), pT 3 - 4, pN 2 can be given priority to comprehensive treatment including lateral lymph node dissection. Other low-risk factors can be carefully performed lateral lymph node dissection to avoid complications and trauma caused by lateral lymph node dissection. There were some limitations in our study. First, most of the articles included were retrospective studies. Second, except for two articles from Western countries (America and Switzerland), the rest were from Eastern countries, which might have caused our research to be slightly biased towards the Eastern perspective.

## 5 Conclusion

Our studies proved that female sex, age <60 years, pretherapeutic CEA level >5 ng/ml, cT4, cM1, distance of the tumour from the AV <50 mm, tumour centre located Rb, SA of LPLN ≥8 mm before nCRT, SA of LPLN ≥5 mm after nCRT, border irregularity of LPLN, tumour size ≥50 mm, pT3-4, pN2, MLNM, LI, VI, CRM (+) and poor differentiation were risk factors for LPLN metastasis. In conclusion, although lateral lymph node dissection can reduce the local recurrence rate, increase the number of lymph nodes harvested, and achieve more accurate assessment of rectal cancer, it also has the risk of increasing surgery-related complications. Whether to perform lateral lymph node dissection in clinical practice can be judged in combination with the above risk factors so that patients with rectal cancer who need lateral lymph node dissection can be accurately screened out to reduce the risk of unnecessary surgical trauma. According to the risk factors of lateral lymph node metastasis, we can adopt different comprehensive treatment strategies. High-risk patients can perform lateral lymph node dissection to effectively reduce local recurrence; In low-risk patients, we can avoid overtreatment, reduce complications and trauma caused by lateral lymph node dissection, and maximize patient survival and quality of life.

## Data availability statement

The raw data supporting the conclusions of this article will be made available by the authors, without undue reservation.

## Author contributions

D-xZ, ZY, and LT acquisition of data, analysis and interpretation of data, and drafting the article; M-nR and Z-lL collect and organize data, and revising the article; J-wX conception and design of the study, and critical revision. All authors contributed to the article and approved the submitted version.
