# Supplementary material for: Risk factors for lateral pelvic lymph node metastasis in patients with lower rectal cancer: a systematic review and meta-analysis
Source: Front Oncol. 2023 Sep 6;13:1219608. doi: 10.3389/fonc.2023.1219608 (PMC10512344; doi:10.3389/fonc.2023.1219608)
Supplement: Supplementary file 1 [file Presentation_1.pptx]

## Slide 1
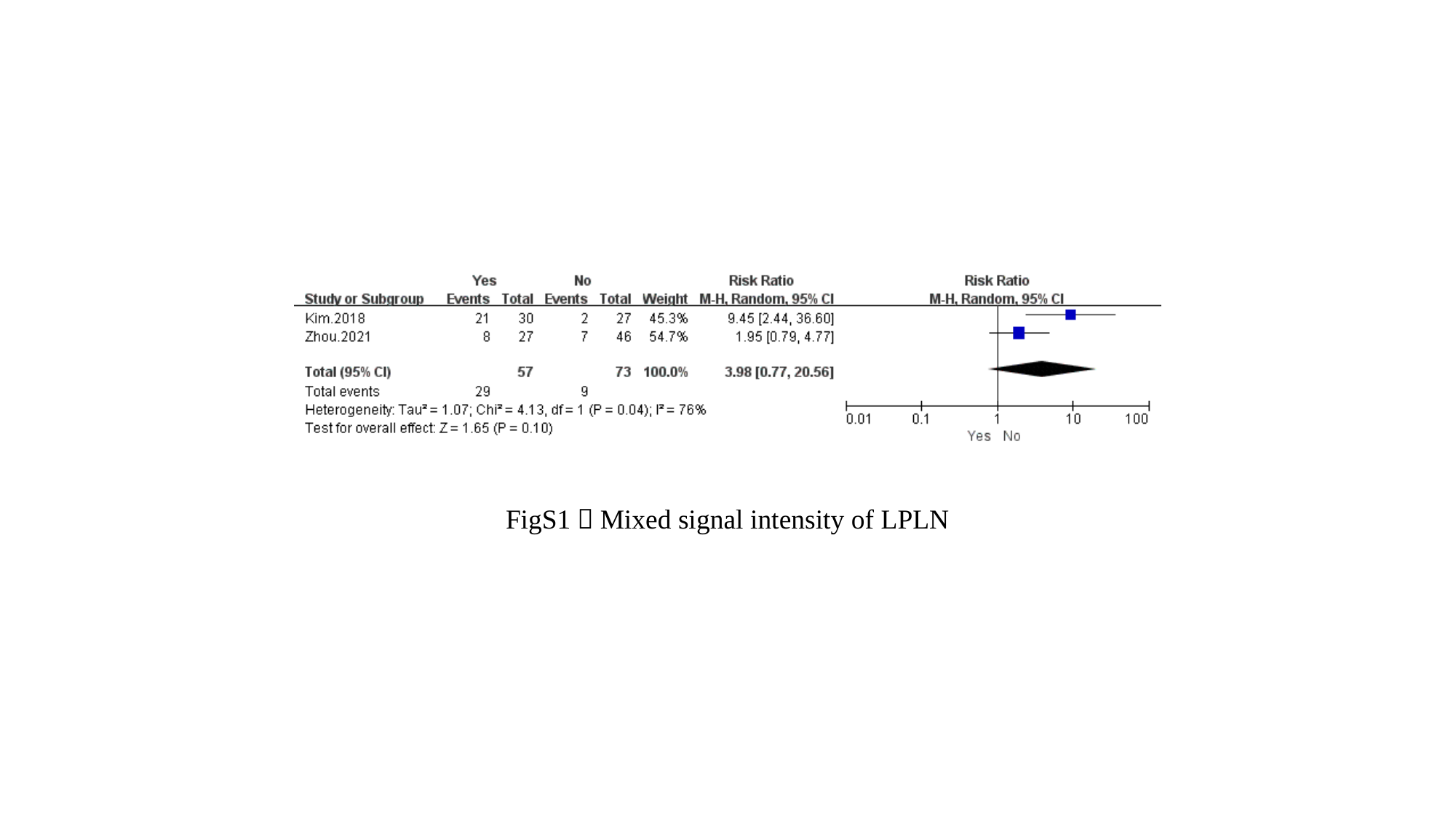

FigS1：Mixed signal intensity of LPLN

## Slide 2
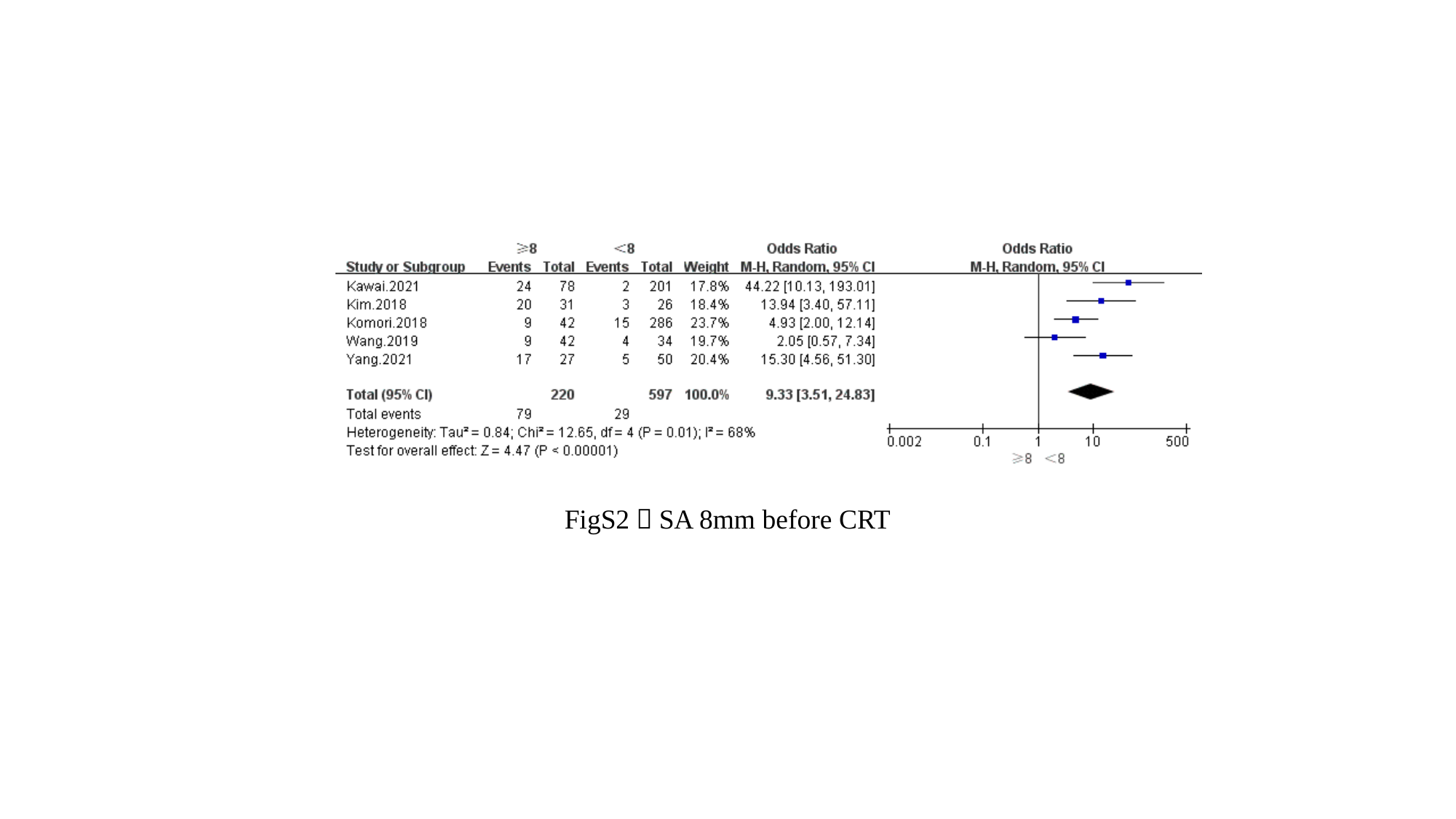

FigS2：SA 8mm before CRT

## Slide 3
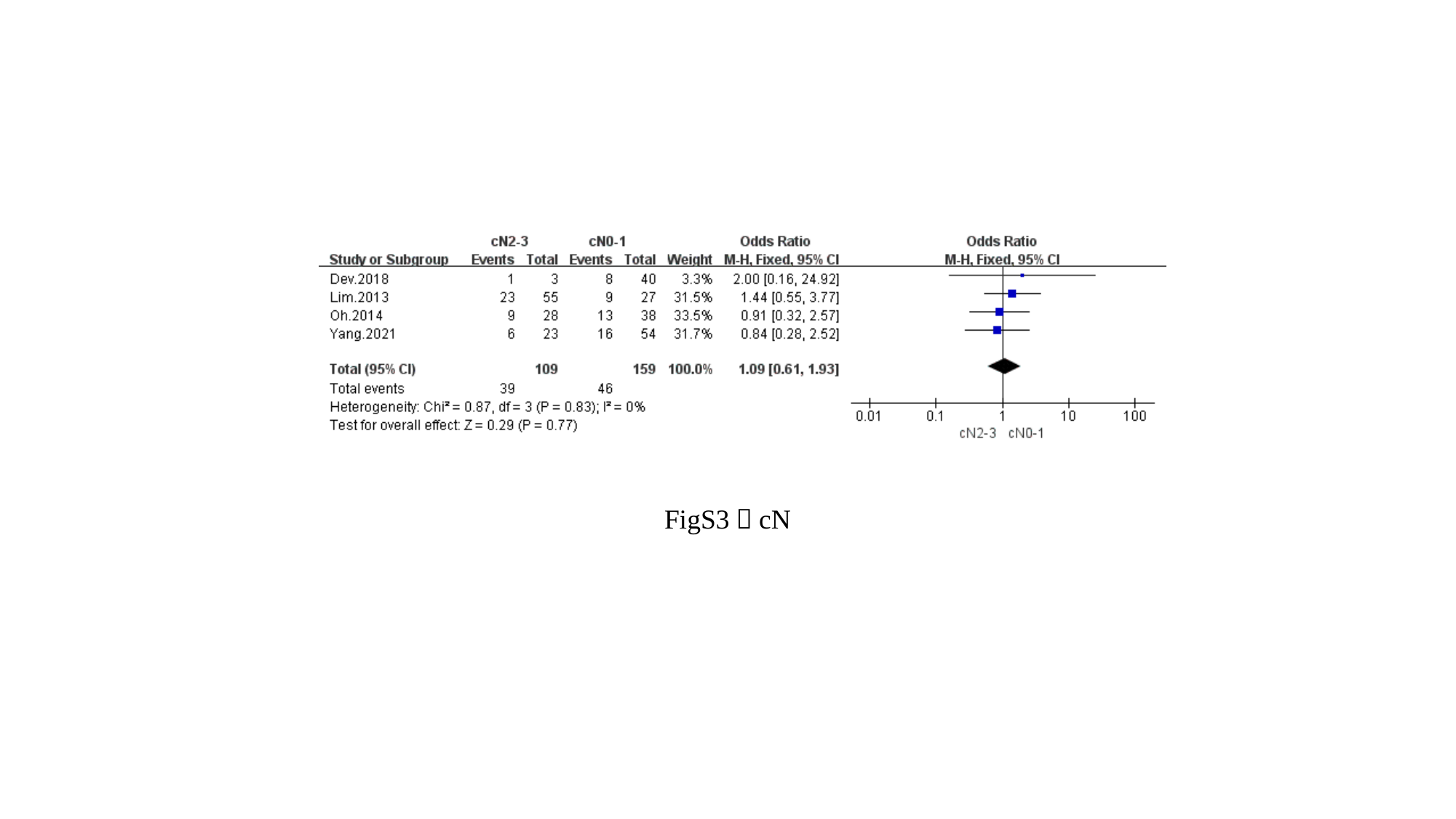

FigS3：cN

## Slide 4
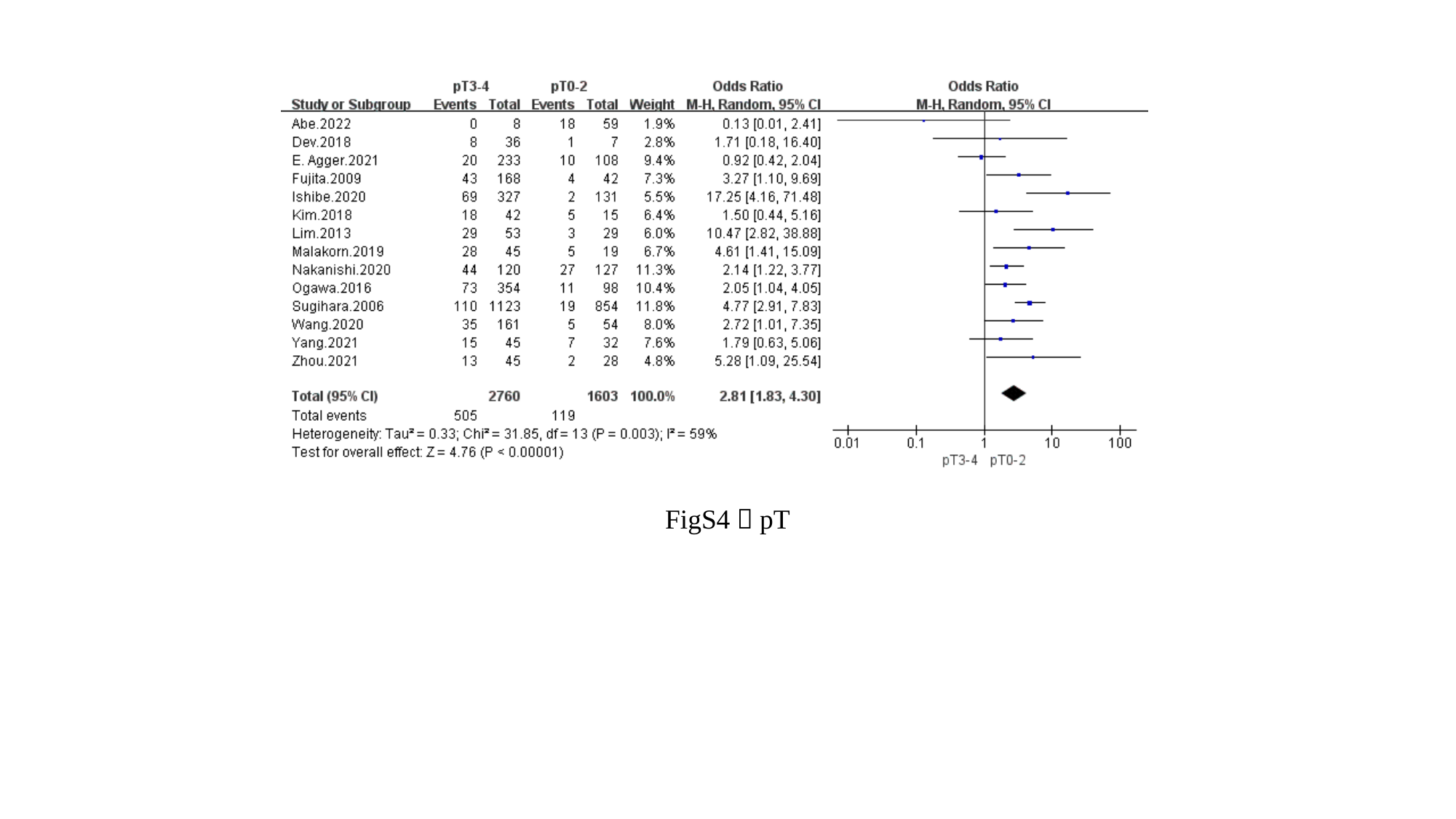

FigS4：pT

## Slide 5
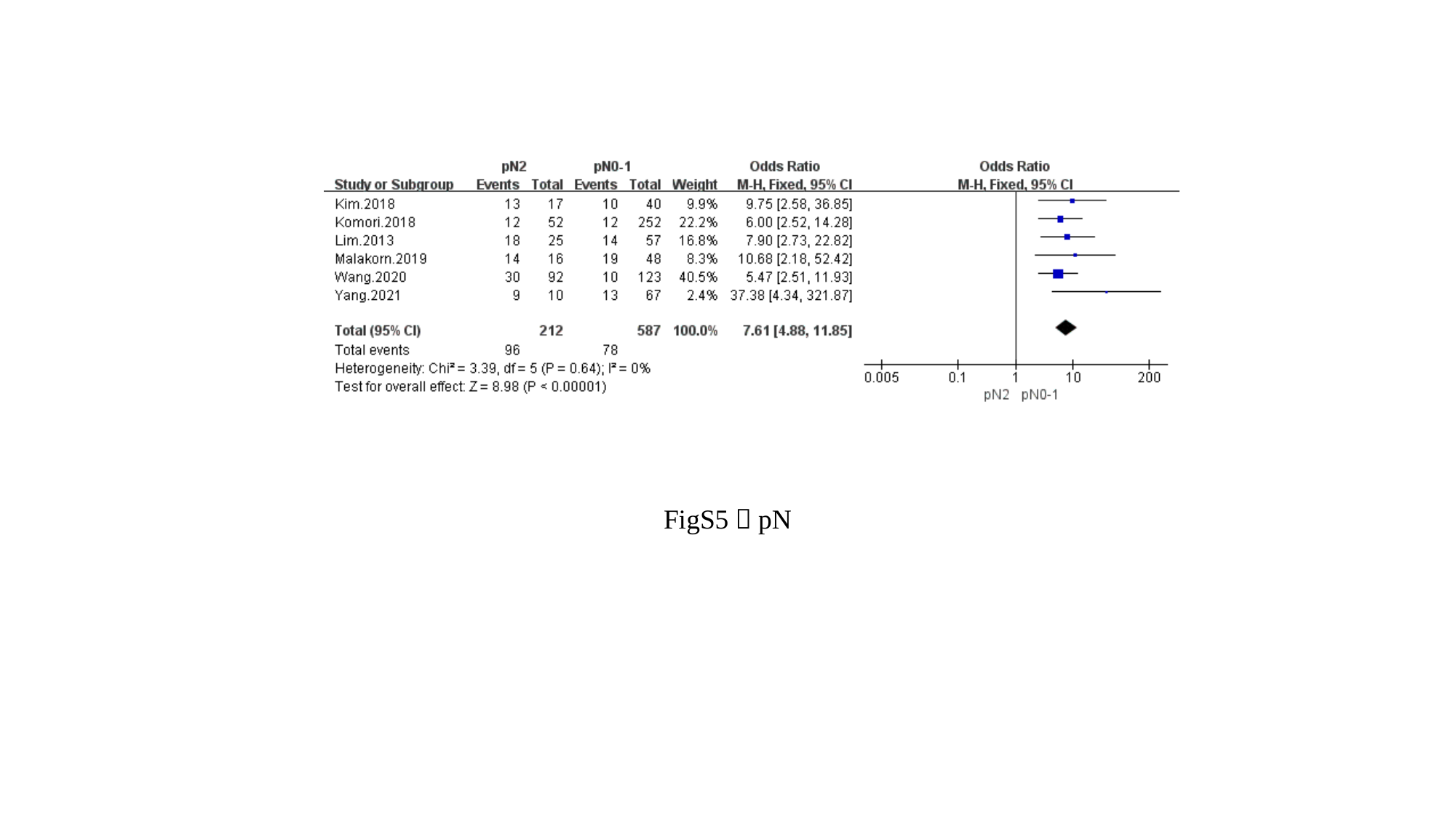

FigS5：pN

## Slide 6
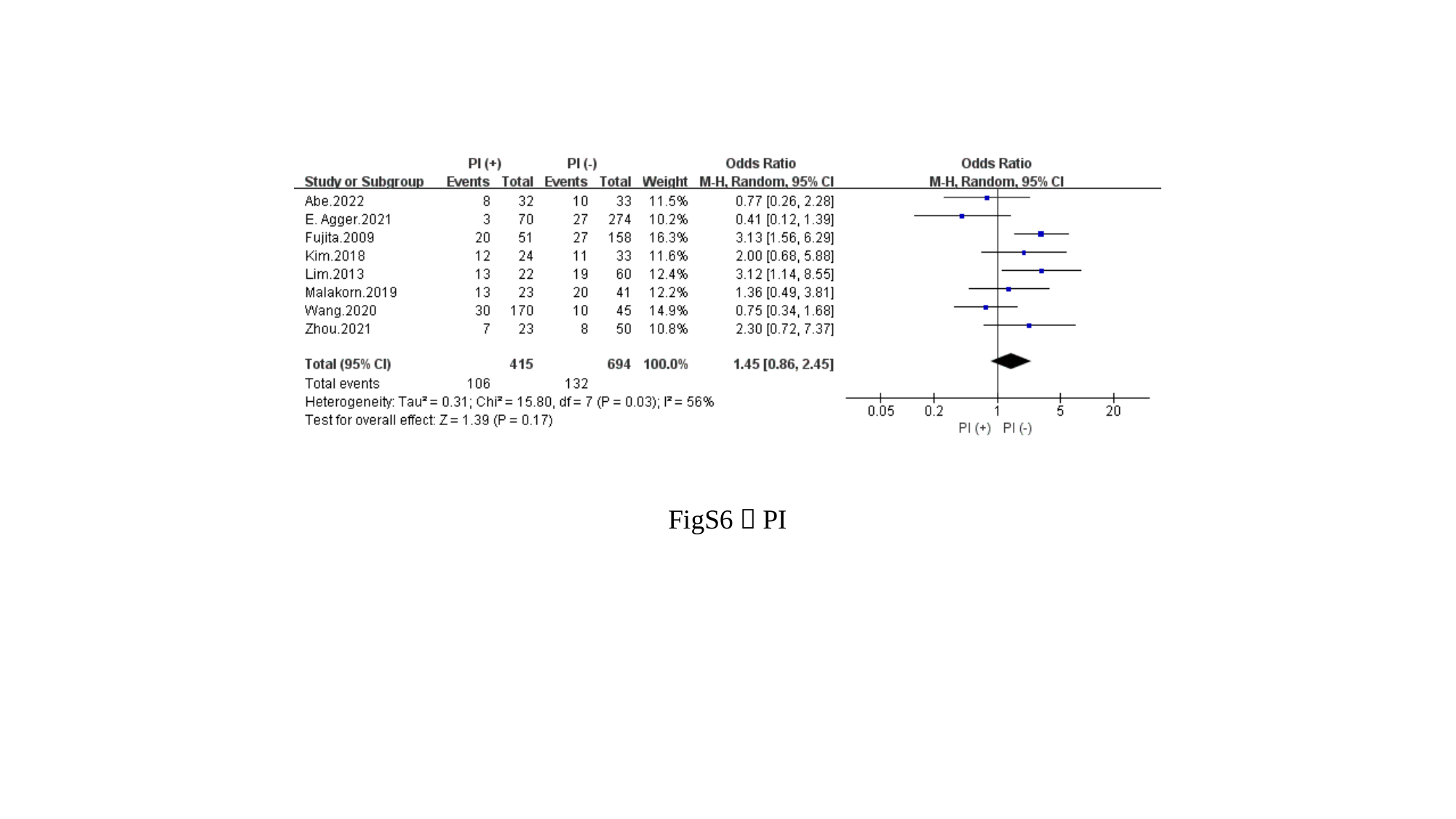

FigS6：PI
